# Identification and verification of seed development related miRNAs in kernel almond by small RNA sequencing and qPCR

**DOI:** 10.1371/journal.pone.0260492

**Published:** 2021-12-01

**Authors:** Marjan Jafari, Behrouz Shiran, Gholamreza Rabiei, Roudabeh Ravash, Badraldin Ebrahim Sayed Tabatabaei, Pedro Martínez-Gómez

**Affiliations:** 1 Department of Horticulture, Faculty of Agriculture, Shahrekord University, Shahrekord, Iran; 2 Department of Plant Breeding and Biotechnology, Faculty of Agriculture, Shahrekord University, Shahrekord, Iran; 3 Institute of Biotechnology, Shahrekord University, Shahrekord, Iran; 4 Department of Biotechnology, College of Agriculture, Isfahan University of Technology, Isfahan, Iran; 5 Department of Plant Breeding, CEBAS-CSIC, Murcia, Spain; Universidade de Lisboa Instituto Superior de Agronomia, PORTUGAL

## Abstract

Many studies have investigated the role of miRNAs on the yield of various plants, but so far, no report is available on the identification and role of miRNAs in fruit and seed development of almonds. In this study, preliminary analysis by high-throughput sequencing of short RNAs of kernels from the crosses between almond cultivars ‘Sefid’ × ‘Mamaee’ (with small and large kernels, respectively) and ‘Sefid’ × ‘*P*. *orientalis*’ (with small kernels) showed that the expressions of several miRNAs such as *Pdu-miR395a-3p*, *Pdu-miR8123-5p*, *Pdu-miR482f*, *Pdu-miR6285*, and *Pdu-miR396a* were significantly different. These miRNAs targeted genes encoding different proteins such as *NYFB-3*, *SPX1*, *PGSIP3 (GUX2)*, *GH3*.*9*, and *BEN1*. The result of RT-qPCR revealed that the expression of these genes showed significant differences between the crosses and developmental stages of the seeds, suggesting that these genes might be involved in controlling kernel size because the presence of these miRNAs had a negative effect on their target genes. Pollen source can influence kernel size by affecting hormonal signaling and metabolic pathways through related miRNAs, a phenomenon known as xenia.

## Introduction

The almond (*Prunus dulcis* [Mill.] D. A. Webb) belongs to the family Rosaceae. It is commercially grown for its nutritious kernels [1]. The edible part of the almond fruit is the kernel (seed), thus fertilization of the ovary is necessary [2]. Many commercial almond cultivars are self-incompatible and need a compatible pollinizer to set fruit [3, 4]. Almond growers need to select at least two cross-compatible cultivars with sufficient overlapping of their flowering time to reach a higher economical yield [5]. It has been reported in many studies that seed quantity and quality of some plant species such as almonds [6], and hazelnuts [7, 8] might be affected by pollinizers genotypes, a phenomenon known as xenia effect. In almonds, pollination with sweet kernel cultivars has increased the size of the kernel compared to bitter kernel cultivars [9]. Fertilization trials with pollens from a large-seeded cultivar or species of pistachio or almond resulted in larger kernels on mother plants that otherwise would produce smaller seeds [10, 11]. Recently, our transcriptomic analysis has identified some functional genes such as SUS2, GASA1, BEN1, CYP72C1 ABCG25 and GA2OX2 responsible for seed size in developing kernels of almond [11].

Plants have attained highly regulated gene expression patterns to guarantee proper development and function of tissues and responses to environmental cues. Since gene expression is a multistep process, it can be regulated at several levels. Small RNA molecules (sRNA) has been known as a post-transcriptional gene regulatory mechanism [12]. A microRNA (miRNA) is the final product of a non-coding RNA gene that is small RNA molecules with 21–24 bases in length. The mature miRNA is complexed with a ribonucleoprotein named RISC that can base-paired with its target genes, where it post-transcriptionally regulate the expression of its target mRNA using the cleavage or translational repression mechanism [13]. Most miRNAs negatively regulate their target genes therefore when both are expressed in the same cells, and their expression level change over time a negative correlation can be observed [14, 15].

Plant microRNAs are characterized as having a high degree of sequence complementarity to their target mRNAs and have been predicted or confirmed to control the regulation of genes encoding several types of proteins. The transcription factors or signal transduction or other regulatory proteins that function in plant development are the major classes of miRNA target genes [16]. MicroRNAs and their targets encoding functional genes and transcription factors are, to a major extent, involved in the seed development and maturation process. The involvement of miRNAs in post-transcriptional regulation of seed or fruit development has been documented in some crops. For example; the role of miRNA in seed development was investigated by performing an integrated analysis of mRNA and miRNA transcriptome as well as RT-qPCR detection in Siberian apricot [17]. Some key miRNAs and their targets were identified that could potentially involve in developing response and hormone signaling of kernels including *miR160*-ARF18, *miR156*-SPL, *miR171h*-SCL6, *miR164*-NAC1, *miR172*-AP2, *miR393h*-TIR1/AFB2, *miR395*-AUX22B, *miR530*-P2C37, and *Psi-miRn5*-SnRK2A. In pear, Wu et al. [18] showed the role and changes of *miR156k*, *miR159b-3p*, *miR164c*, *miR166a*, *miR396b-3p*, and *miR4376-5p* during fruit development. Yao et al. [19] showed that the expression level of a *miRNA172* and its target gene APETALA2 (AP2) influences fruit size in apples. It was shown that the over-expression of *miRNA172* in transgenic apple significantly reduced both the AP2 gene expression and fruit size [19]. It is well known that *miRNA172* inhibits AP2 translation in Arabidopsis and increases its seed size [16]. Similarly, Allen et al. [20] were double-mutated the *miR159* gene (*miR159ab*) in Arabidopsis, causing an increase in MYB33 and MYB65 expression levels that led to the formation of small seeds. Zhang et al. [21] reported that Cme-miR164c/d were up-regulated during fruit development in Hami melon indicating miR164 through regulating NAC transcription factors and EVE1 may negatively regulate cell proliferation and expansion at the early stages of fruit development [21]. The overexpression of *Os-miR397* in rice, down regulated its target gene, OsLAC, whose product is a laccase-like protein that is involved in plants sensitivity to brassinosteroids, improves rice yield through increasing grain size and promoting panicle branching [22]. Fruit size and weight of tomatoes was negatively regulated by miR396 via controlling Growth Regulating Factors (GRFs) transcription factors [23]. Zheng et al [24] revealed a miR164-dependent regulatory pathway, miR164-NAC32/NAC40-EXPB14/EXPB15, which participates in maize seed expansion. Dhaka and Sharma [25] identified 21 different miRNAs experimentally to regulate seed size, nutritional content, vigor, and shattering. Moreover, high throughput sequencing has been recently used for the identification and expression profiling of miRNAs in many fruit crops, such as sweet orange [26], grape [27], papaya [28], strawberry [29, 30], olive [31], banana [32] and Grapevine [33].

Kernel size in almond is regarded as an important quality parameter. While, there are information about the role of miRNAs in controlling seed size in different species, there are no reports on miRNAs involvement in kernel development of almonds. To understand the regulation of seed size and investigating the paternal effects, the present study was carried out to identify miRNAs and their target genes involved in developing seeds of almond. For this purpose, four almond cultivars with different seed sizes were selected and different crosses were performed between them. The resulting seeds were collected at several stages of fruit development and were used for small RNA (sRNA) sequencing and qPCR. An integrated analysis of miRNA transcriptome and RT-qPCR identified some miRNAs and their targets potentially involved in controlling almond seed size.

## Materials and methods

### Plant materials, pollination and sampling

Two cultivars were chosen as female parents to perform the crosses, including ‘Sefid’ and ‘Mamaee Pooya’ with small and large seed sizes, respectively. Each cultivar was hand-pollinated with two pollen types: ‘Mamaee’ (with a large kernel) and a close species ‘*P*. *orientalis*’ (with a small kernel). This experiment was carried out in an almond orchard of ZayandehRood Horticultural Company, Chaharmahal and Bakhtiari Research and Technology Park located in Shoorab-e-Saghir village (32°30’42.7"N 50°56’20.5"E) in Saman County, Chaharmahal and Bakhtiari province, Iran, in a factorial experiment using a randomized complete block design with two replications and four crosses. The crosses included of ‘Sefid’ (♀ parent) × ‘Mamaee’ (♂ parent) (SM), ‘Sefid’ (♀ parent) × ‘Orientalis’ (♂ parent) (SO), ‘Mamaee Pooya’ (♀ parent) × ‘Mamaee’ (♂ parent) (PM), ‘Mamaee Pooya’ (♀ parent) × ‘Orientalis’ (♂ parent) (PO). Each replication included one tree and two branches on two sides of the tree (North and South) were selected for each cross. Single composite sample of two biological replicate was prepared from each of SO and SM crosses and used for a preliminary small RNA sequencing [34, 35]. To verify the small RNAseq results, we were used RNAs samples of two biological replicates and two technical replicates by qPCR in four crosses (SO, SM, PO and PM). Also, two biological replicates and two technical replicates of the five developmental stages (0, 18, 36, 61 and 72 days after pollination) were used to study the expression pattern of miRNA and their target genes. Two crosses including ‘Sefid’ (♀) × ‘Mamaee’ (♂) (SM) and ‘Sefid’ (♀) × ‘*P*. *orientalis*’ (♂) (SO) were used for sRNA sequencing. In addition, four crosses of ‘Sefid’ (♀) × ‘Mamaee’ (♂); ‘Sefid’ (♀) × ‘*P*. *orientalis*’ (♂); ‘Mamaee Pooya’ (♀) × ‘Mamaee’ (♂) and ‘Mamaee Pooya’ (♀) × ‘*P*. *orientalis*’ (♂), were used to confirm the sRNAseq results and also studying the expression patterns of some miRNA in different developmental stages through RT-qPCR [36]. This experiment was carried out in an almond orchard of ZayandehRood Horticultural Company, Chaharmahal and Bakhtiari Research and Technology Park located in Shoorab-e-Saghir village (32°30’42.7"N 50°56’20.5"E) in Saman County, Chaharmahal and Bakhtiari province, Iran, in a factorial experiment using a randomized complete block design.

Healthy and uniform branches were selected for hand pollinations to identify miRNA and target genes related to kernel size through the analysis of the effect of pollen. Flowers of the female parents were emasculated at the popcorn stage and were hand pollinated using the pollen that had been collected and dried at room temperature and kept in small glass vials at 4°C. Immature seeds at several developmental stages were sampled including 0, 18, 24, 30, 36, 61, and 72 days after pollination (DAP) and were immediately frozen in liquid nitrogen and later stored at -80°C until used. All the samples were collected in March-June 2018.

### RNA extraction and sequencing

100 mg of immature kernels were sampled for total RNA extraction according to the protocol described by Rubio-Piña and Zapata-Pérez. [37]. Samples were powdered using liquid nitrogen and 1 ml extraction buffer containing 0.1 M Tris-HCl (pH 8); 20 mM EDTA (pH 8); 1.4 M NaCl; 2% (w/v) CTAB; 2% (w/v) PVP and 70 μL of β-mercaptoethanol was added to each sample in 2 mL microfuge tube. Then samples were placed in thermal block set at 65°C for 10 min. Afterwards, 800 μL chloroform was added per each sample and briefly shacked, then centrifuged at 10,000 rpm at 4°C for 10 min. The supernatant was extracted twice with an equal volume of phenol/chloroform (1:1) and chloroform/isoamyl alcohol (24:1), respectively. Finally, LiCl (8M) was added to each tube and placed at -20°C for 4h, then centrifuged. Total RNA pellets were washed and after drying, dissolved in DEPC water. The quantity and quality of isolated RNA were verified using Biophotometer Eppendorf and RNA integrity assessed using non-denaturing agarose gel electrophoresis. To perform small RNA sequencing, the RNAs obtained from different stages of seed development from the ‘Sefid’ × ‘Mamaee’ (SM) and ‘Sefid’ × ‘*P*. *orientalis*’ (SO) that had a different size in final stage (mature seed) were mixed together to form two composite samples, respectively. The quality of RNAs were checked using an Agilent Technologies 2100 Bioanalyzer with an RNA Integrity Number (RIN) value of 8.3 and 8.4 for SM and SO, respectively. cDNA libraries were constructed using TruSeq Small RNA Library Prep kit and sequencing were carried out by Macrogen company, Korea using Illumina HiSeq 2000 platform. Raw data produced in FASTQ format and obtained from the company’s server. The raw sRNA data has been deposited in the sequence reads archive (SRA), NCBI, and could be accessed using Bioproject number PRJNA673321.

### Identification of conserved miRNAs in developing seed of the almond

The identification and analysis of raw reads were carried out using the procedure described by Barakat et al. [38] with some modification. The quality control (QC) performed using SolexaQA software followed by the extraction of sequences between 18 and 24 base pairs using an in-house Perl script [39]. sRNA sequences that passed the size filter using BLASTN program, were then queried against rfam (http://rfam.sanger.ac.uk/), chloroplast and mitochondrial genomes (http://gobase.bcm.umontreal.ca/), tRNA (http://www.psb.ugent.be/rRNA/), rRNA (http://lowelab.ucsc.edu/GtRNAdb/), snoRNA (http://bioinf.scri.sari.ac.uk/cgibin/plant_snorna/home/) and all contaminating tRNA, rRNA and snoRNA sequences were removed. Unique sRNA sequences were blasted against known miRNAs (http://www.miRbase.org/index.shtml) to recognize conserved miRNAs, using default parameters that allowed up to two mismatches.

### Differential expression analysis of miRNAs related to kernel size

The counts of identified miRNAs were normalized in two libraries as counts per million (CPM) according to the formula: Normalized expression = actual miRNA count/total count of clean reads × 1,000,000. The normalized reads were used to calculate p-value and Fold-change in expression abundance. The differential expression of miRNAs between 2 libraries was calculated as Fold-change = log2 (value SM/value SO). The miRNAs with absolute values of log2 (ratio) > 0.5, along with p-value < 0.05, were considered with significantly differential expression [38]. The p-value was obtained according to Gao et al. [40].

### Identification of target genes for differentially expressed miRNAs

The miRNAs that revealed significant differential expression (p<0.05) between the two SM and SO samples were submitted to the psRNATarget web server (http://plantgrn.noble.org/psRNATarget/) with default parameters to predict the potential targets for each miRNA [41]. psRNATarget server allows to reverse complementary matching between a miRNA and its target transcript. In order to identify almond specific target genes, which are under control by conserved miRNAs, we have used almond genome (http://ftp.ebi.ac.uk/ensemblgenomes/pub/release-51/plants/fasta/prunus_dulcis/). For annotation of the target genes we have used Arabidopsis protein database which includes functional annotation of the predicted genes. The interaction networks of miRNAs with target genes were constructed using Cytoscape software version of 3.7.2.

### Functional enrichment analysis for differential miRNAs’ target transcripts

In order to know the function of the differentially expressed miRNA, GO and KEGG pathway enrichment analysis of the target genes was carried out by AgriGO (http://bioinfo.cau.edu.cn/agriGO/) [42]. This method maps all target gene candidates to calculating gene numbers for each GO term and pathway in this server.

### Evaluation of miRNAs expression and their potential target genes through qPCR analysis

The expression profiles of five mature miRNAs that show the different expression in two libraries confirmed using stem-loop reverse transcription-PCR (RT-PCR) in four pooled samples of SO, SM, PO and PM. Also, the expression of miRNAs and their target genes examined in different developmental stages including 0, 18, 36, 61 and 72 days after pollination. The stem-loop RT-PCR method was performed as described by Varkonyi-Gasic et al. [43] and explained in Karimi et al. [44]. Briefly, for each miRNA, cDNA was synthesized using 200 ng DNase I-treated total RNA which reverse transcribed using M-MuLV Reverse Transcriptase (YTA, Cat No: YT4500) and stem-loop RT-primers specific to each miRNA. Reverse transcription was performed at 16°C for 30 min followed by 42°C for 30 min and then 85°C for 5 min. miRNA primer designer software (http://genomics.dote.hu:8080/mirnadesigntool/) was used to design the primers. For target genes, 2 μg DNase I-treated total RNA was used to synthesize first-strand cDNA by M-MLV reverse transcriptase using oligo(dT) primer. Reverse transcription was performed at 42°C for 60 min followed by 85°C for 5 min, and then held at 4°C. The qPCR reaction was performed in a total volume of 12 μL using a micPCR (BioMolecular Systems) instrument. Each reaction contained 1 μL of cDNA, 6 μL SYBR green PCR master mix (YTA), 1 μM of each forward and reversed primers. 18 S and α-tubulin genes were used as the internal control. Two biological repeats and two technical replicates were performed to analyses expression patterns of each miRNA and their target genes. The relative expression changes of mature miRNAs and target genes were calculated using the 2^−ΔΔCT^ method. The analysis of variance was conducted on RT-qPCR results using general linear model (GLM) procedure of SAS software, arithmetic means were compared by the Duncan multiple range test (p < 0.05). The list of all the Primers were used in quantitative RT-PCR experiments are in S1 Table.

## Result

### High-throughput sequencing of small RNAs libraries in almond

To identify miRNA-mediated regulation that may be involved in almond kernel size, two composite small RNA libraries were constructed from immature seeds of the crosses SM (large seed) and SO (small seed). A total of 42,368,292 and 47,951,632 raw reads were obtained for SM and SO, respectively from the small RNA library using the Illumina sequencing platform with an average read length of 51 nucleotides. GC contents were 51.63% and 51.97% for SM and SO, respectively (Table 1).

**Table 1:** The total number of bases, reads, GC (%), Q20 (%), and Q30 (%) are calculated for the 2 assayed almond cultivars.

Sample ID	Total read bases(bp)	Total reads	GC(%)	AT(%)	Q20(%)	Q30(%)
**SM**	2,160,782,892	42,368,292	51.63	48.37	97.23	94.32
**SO**	2,445,533,232	47,951,632	51.97	48.03	97.18	94.39

• Sample ID: Sample name. SM, the cross between ‘Sefid’×‘Mamaee’; SO the cross between ‘Sefid’×‘*P*. *orientalis*’.

• Total read bases: Total number of bases sequenced.

• Total read: Total number of reads. For illumine paired-end sequencing. This value refers to the sum of read 1 and read 2.

• GC(%): GC content.

• AT(%): AT content.

• Q20(%): Ratio of bases that have phred quality score of over 20.

• Q30(%): Ratio of bases that have phred quality score of over 30.

### Identification of conserved miRNAs in almond

**Fig 1:**
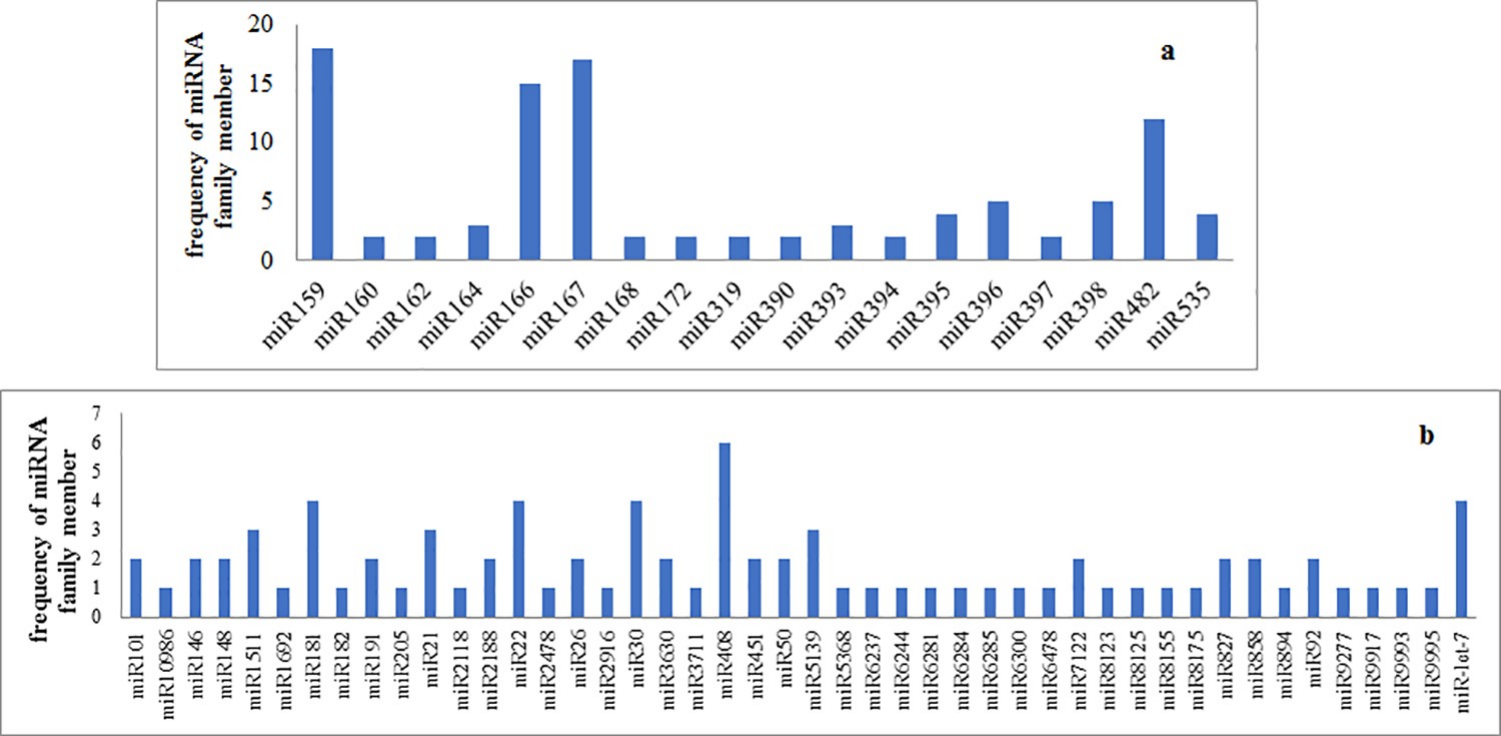
The distribution of known miRNA families in almond. a: Highly conserved; b: Conserved in some species.

After sequence analysis of sRNA libraries using a blast against miRBase database (version 20), sequence similarity discovered the existence of 178 and 174 miRNAs in the SO and SM libraries. Total of 64 miRNA families were identified unique in both libraries out of which, uniquely 18 and 46 families were highly conserved and conserved in some plant species, respectively (S2 Table). The highly conserved miRNA families included 2–18 members in our dataset. Size distribution of highly conserved miRNAs revealed that *miR159*, *miR167*, *miR166*, and *miR159* families had the highest number of miRNAs (Fig 1A). However, among the conserved miRNAs family, *miR408*, miR30, *miR22* and *miR181* had the highest number of members, while the most of the other conserved miRNA families composed of only one member (Fig 1B).

**Fig 2:**
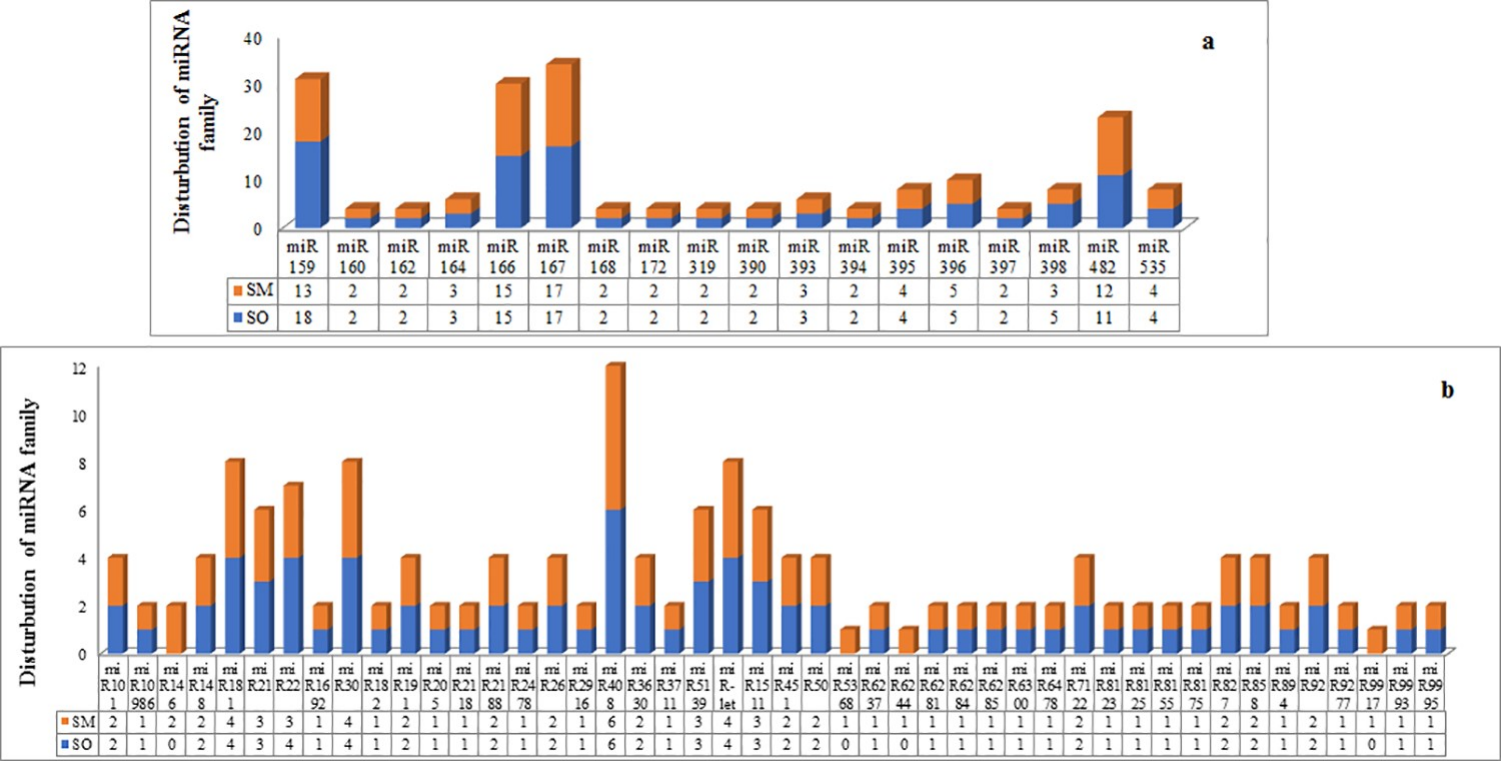
The distribution of known miRNA families in SO and SM libraries. a: Highly conserved; b: Conserved in some species.

Also, the size distribution of miRNA families between the two libraries recognized 58 common miRNAs, though the number of members were different in each library (Fig 2A and 2B).

### Differential expression analysis of miRNAs in developing seeds

**Fig 3:**
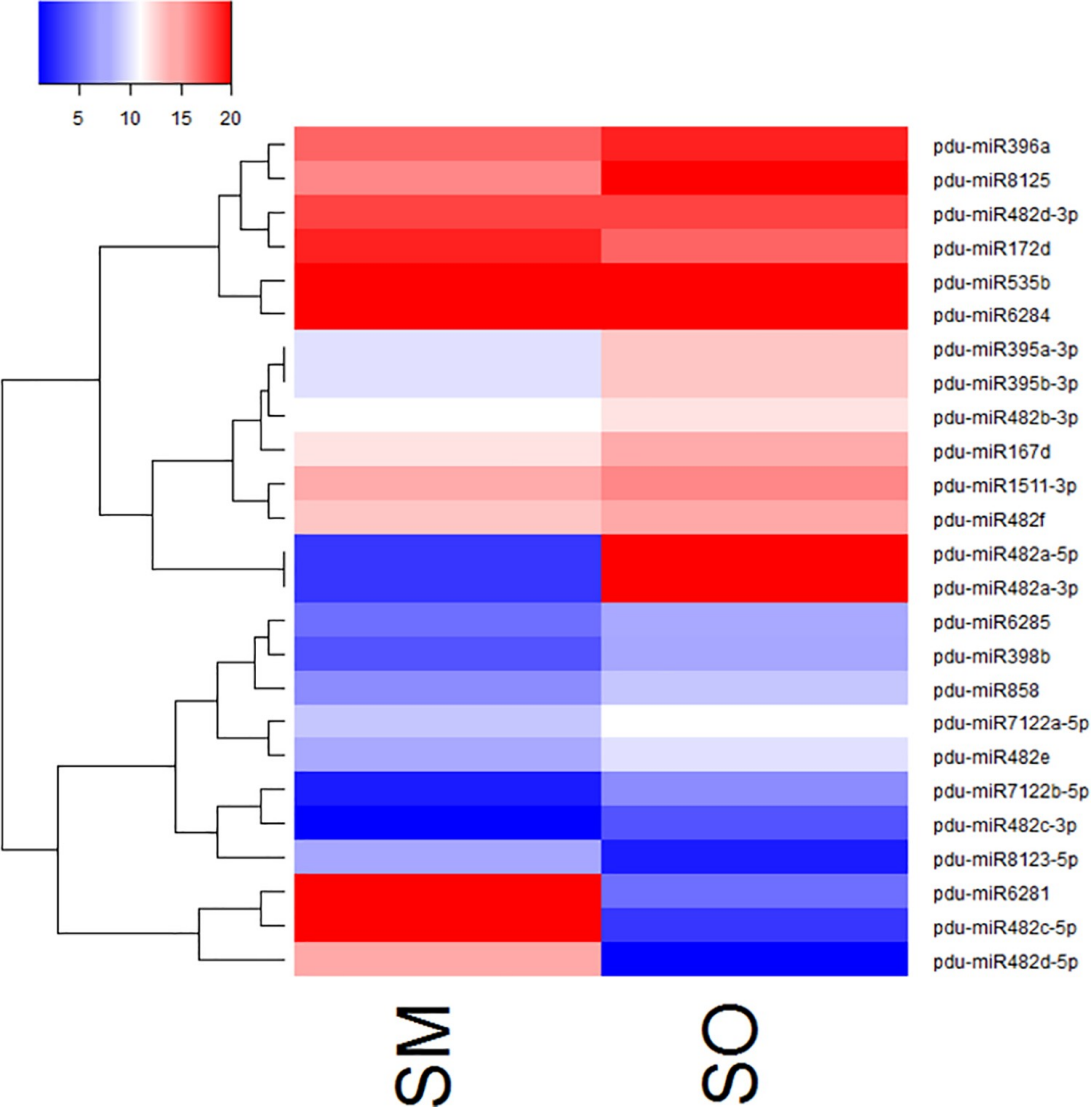
The heatmaps of differentially expressed miRNAs in developing seeds of SM and SO. The color shows the log 2 CPM of miRNAs expression among the SM and SO. As the color gets closer to bright red, increased the expression of miRNA.

Expression analysis of conserved miRNAs showed that *miR482*, *miR1511*, and *miR167* families had the highest expression in the two libraries. However, distinct expression variation was observed within the members of some families. For example, *miR482b-3p* had 9973 count per million (CPM) in SM library, whereas the read number for *miR482d-3p* was only 254 CPM. Interestingly, one miRNAs were found to be expressed solely in the SM cross (Fig 3).

### Identification of target genes for differentially expressed miRNAs

**Fig 4:**
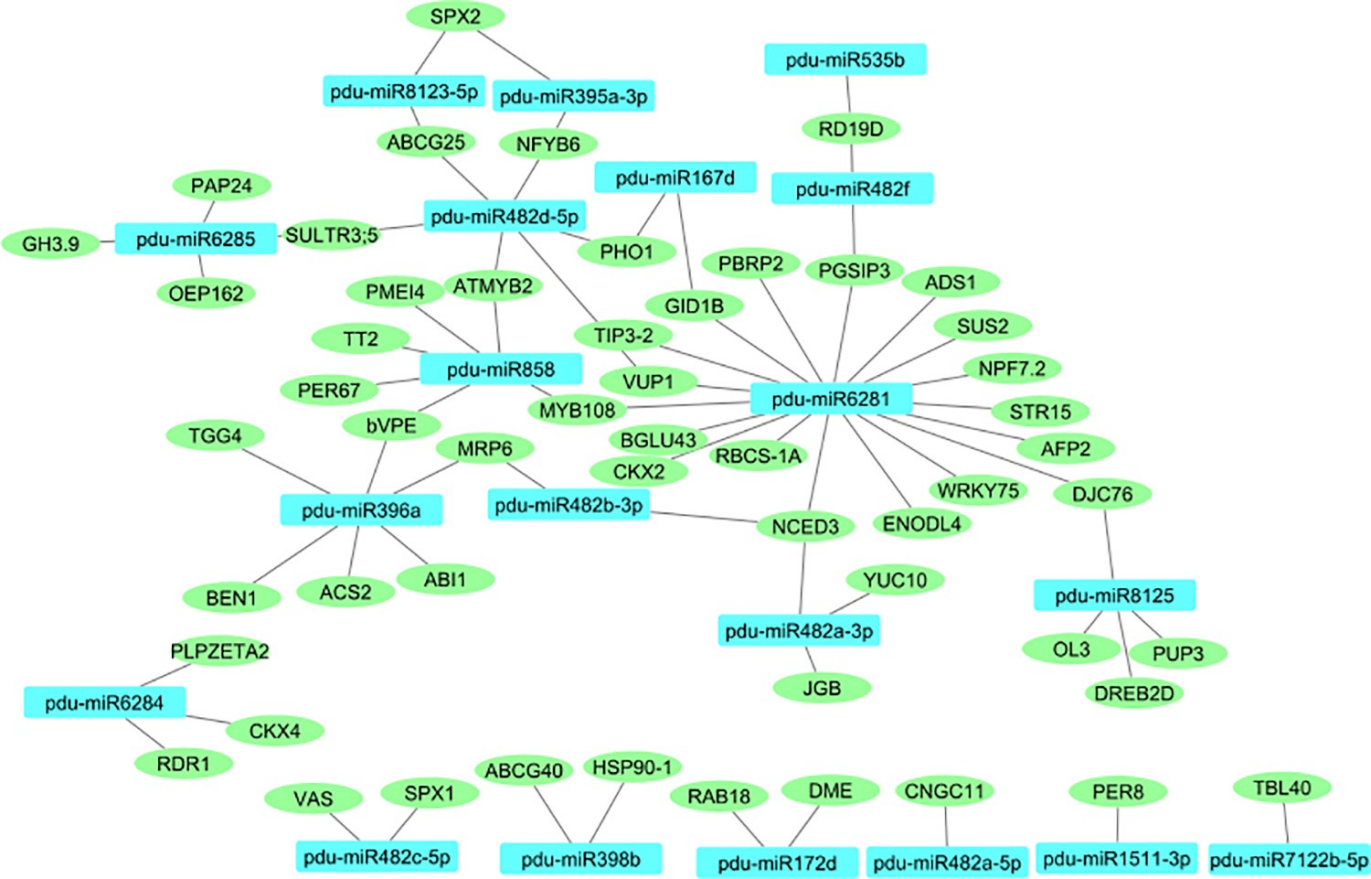
Interaction network of DE miRNAs and their target genes involved in seed size of almond. Rectangles and circles represented miRNA and target genes, respectively.

In the present study, we have identified the miRNAs that displayed differential expression in various seed sizes of *Prunus dulcis*. In order to study the role of these miRNAs, we used psRNA Target, a web-based program, for the prediction of putative miRNA targets [41]. A total of 73 almond genes were predicted as putative targets of 20 conserved miRNA families. The details of miRNAs and their target genes are available in the S3 Table. The interaction network of miRNAs and target genes is shown in Fig 4. The network comprises 73 nodes. The number of genes targeted by a single miRNA differs from one to eighteen, with a majority of them targeting two genes. *Pdu-miR6281* targets 18 genes.

### Functional enrichment for differential miRNAs’ target genes

**Fig 5:**
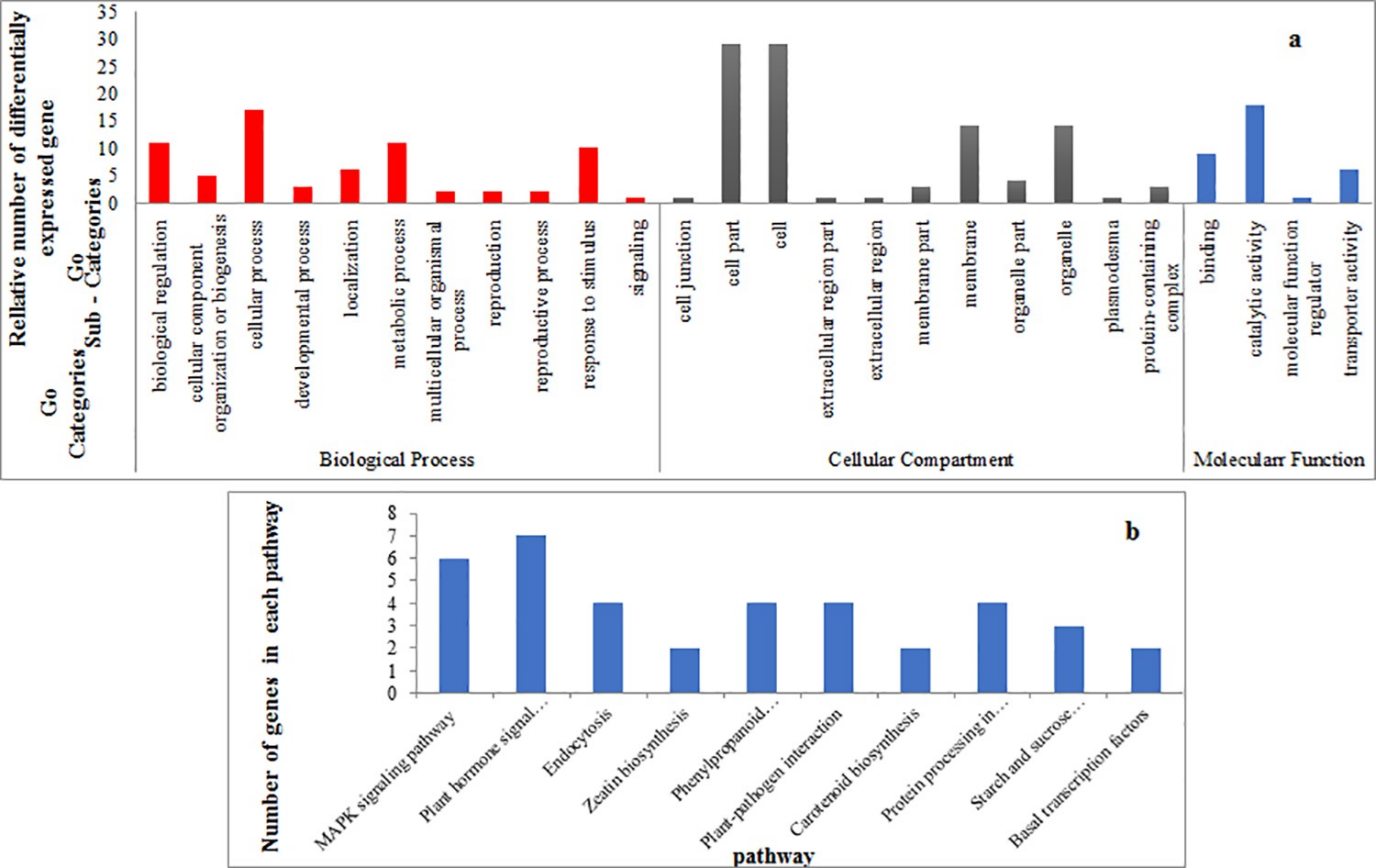
Functional enrichment analysis for differential miRNAs’ target. (a) GO annotation of target genes. Y-axis (right) represents the actual gene number. The genes were annotated in three main categories including biological progress, cellular component, and molecular function (X-axis). (b) Functional annotation of KEGG pathways by KEGG database.

To further elucidate the functional roles of target gene of DEGs miRNAs on kernel size, we performed Gene Ontology (GO) and Kyoto Encyclopedia of Genes and Genomes (KEGG) pathway enrichment analysis for the target gene using gene ontology online tool. The biological processes, molecular functions and cellular components of the targets are shown in Fig 5A. Under biological process, majority of the targets (55.7) are involved in the metabolic, biological regulation, and cellular process. Around 14.3% of the targets are responsible for the response to a stimulus. The remaining (30%) are involved in a plethora of processes including cellular component organization or biogenesis, reproductive process, reproduction, development, localization, multicellular organismal process and signaling. The molecular functions performed by the targets cover almost all aspects of plant metabolism. Majority of the targets perform functions in catalytic activity (52.9%) and binding (26.5%). The remaining 20% involved transporter activity and molecular function regulator. The proteins coded by miRNA targets localize in different cellular components. A large number of proteins localize in cell, cell part, organelle and organelle parts (86%). The remaining (14%) are localized in cell junction, extracellular region part, extracellular region, membrane part, membrane, plasmodesma, and protein-containing complex. The KEGG pathway analysis revealed 10 overrepresented pathways including plant hormone signaling, MAPK signaling pathway and starch and sucrose metabolism (Fig 5B).

### Validation of expression analysis of known miRNAs

**Fig 6:**
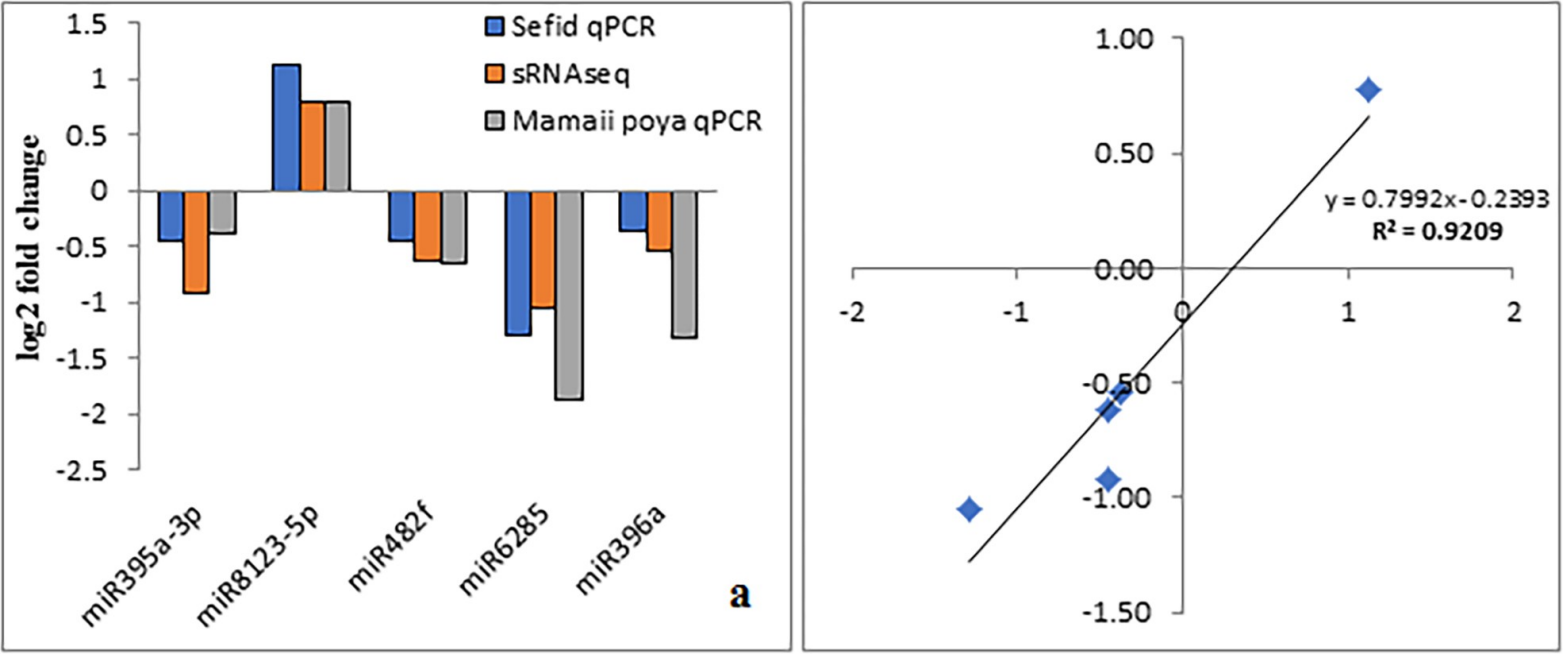
Illustrating of the qPCR confirmation results for the 5 selected miRNAs with differentially expression. (a) The X-axis demonstrate the selected 5 miRNAs and the Y-axis demonstrate the log2 fold change values derived from RNA-seq and qPCR. (b) Regression analysis of the log2 fold change values between RNA-seq and qPCR.

To confirm the expression patterns detected for miRNAs in the high throughput sequencing between SM and SO samples, five differentially expressed miRNAs including *Pdu-miR396a*, *Pdu-miR6285*, *Pdu-miR395a-3p*, *Pdu-miR8123-5p* and *Pdu-miR482f* were preliminary selected and analyzed with qPCR in a pooled sample of four crosses including SM, SO, PM, and PO. The comparison of expression pattern between small RNA sequencing data and qPCR results revealed very similar patterns of expression in seed samples of all crosses for selected miRNAs (Fig 6A). There was a high positive correlation (R^2^ = 0.9209) between the qPCR and small RNAseq log2 fold changes (Fig 6B).

### Expression pattern analysis of known miRNAs and their target genes in seed developmental stages

**Fig 7:**
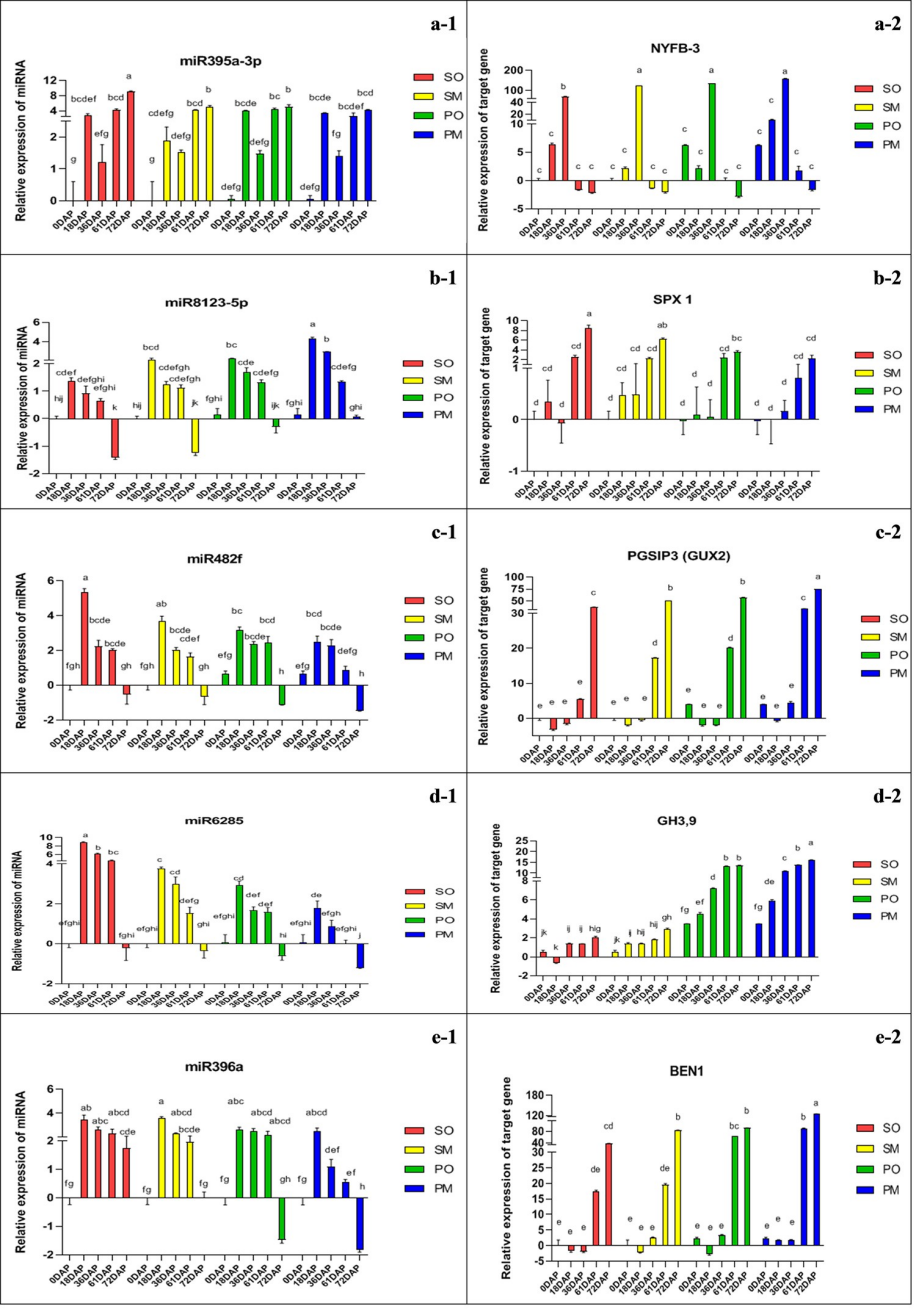
qPCR analysis of kernel size miRNAs and their targets expression profiles in developing kernel of SM, SO, PM and PO almond crosses. Values represent arithmetic mean of two replicates ± SE (standard error). Different lowercase letters indicate significant differences at p < 0.05 in the Duncan’s multiple range test.

We explored the expression pattern of five miRNAs and their putative target genes (*Pdu-miR395a-3p*_ *NYFB-6*, *Pdu-miR8123-5p*_ *SPX2*, *Pdu-miR482f*_ *PGSIP*, *Pdu-miR6285*_ *GH3*.*9*, and *Pdu-miR396a*_ *BEN1)* in five developmental stages of almond including 0, 18, 36, 61 and 72 days after pollination (DAP). The relationship of these miRNAs and their target genes showed an overall negative expression pattern in different stages of fruit development (Fig 7). Given this correlation between miRNAs and target genes as well as the function of target genes in hormone signaling, starch and sucrose metabolism pathways, it could be suggested that these miRNAs involved in kernel developments through these pathways. Statistical analysis carried out to determine the influence of parental and their interaction on expression pattern analysis of five miRNAs and their putative target genes are shown in the S4 Table in the form of the significance of two-way ANOVA test. Based on the results obtained, a significant difference (p<0.05) was observed for the expression of miRNAs and target genes in different crosses and stages of seed development.

The expression pattern of *Pdu-miR395a-3p* is negatively correlated with its corresponding target, Nuclear transcription factor Y subunit B-3 (NYFB-3), a transcription factor involved in seed development which was up-regulated specifically at the final developmental stages of almond kernel in all four crosses (Fig 7A). Based on the results obtained, a significant difference (p<0.05) was observed in the expression of *Pdu-miR395a-3p* in different stages of seed development. The highest and lowest expression of *Pdu-miR395a-3p* were observed 72 days after pollination (DAP) in SO and 0 DAP in SO and SM crosses, respectively. There was no significant difference between 0 DAP all of crosses (Fig 7A-1). The highest and lowest expression of its target gene, NYFB3, was present in 36 DAP and 72 DAP in four crosses, respectively. Also, the PM cross had the highest expression 36 DAP in compared to the other crosses (Fig 7A-2).

Another transcription factor is SPX domain-containing protein 1 (SPX1) that targeted by *Pdu-miR8123-5p*, regulates Pi transport and signaling in the developing kernel of almond and showed a negative correlation with *Pdu-miR8123-5p*. SPX1 was up-regulated specifically at final developmental stages of almond kernel for all crosses (Fig 7B). Significant differences (p<0.05) were found between different crosses as well as developmental stages of each cross for *Pdu-miR8123-5p*. *Pdu-miR8123-5p* had the highest and lowest expression 18 DAP of PM and 72 DAP of SO cross, respectively (Fig 7B-1). 72 DAP and 36 DAP of the SO cross had the highest and lowest SPX1 expression level, respectively. Except for 72 DAP, there was a significant difference between other developmental stages in different crosses (Fig 7B-2).

The transcript of plant glycogenin-like starch initiation proteins (PGSIPs) that are involved in starch synthesis, was negatively correlated with the level of *Pdu-miR482f* and down-regulated at final stages of kernel development (Fig 7C). A significant difference (p<0.05) was observed between different crosses for *Pdu-miR482f* and PGSIP expression, in which the highest and lowest level of *Pdu-miR482f* expressed at 18 DAP in SO and 72 DAP in the PM cross, respectively (Fig 7C-1). Whereas, the PGSIP3 (GUX2) showed the highest and lowest expression in 18 DAP of SM and 72 DAP of the PM crosses, respectively (Fig 7C-2).

Another target involved in hormone signaling was putative indole-3-acetic acid-amido synthetase GH3.9, which connects an amino acid to indole-3-acetic acid (IAA) and lead to the inactivation of the major auxin plant growth regulator, therefore contributes to regulate the level of IAA at late developmental stages. GH3.9 showed a negative correlation with its corresponding miRNA (*Pdu-miR6285*) (Fig 7D), which showed significant differences between different developmental stages. 18 DAP of SO and 72 DAP of the PM crosses showed the highest and lowest level of *Pdu-miR6285* expression, respectively (Fig 7D-1). While the level of GH3.9 expression was the highest and the lowest in 72 DAP of PM and 18 DAP of SO crosses, respectively. ‘Sefid’ in two crosses of *P*. *orientalis* and Mamaee showed lower expression than ‘Mamaee Pooya’ within four crosses examined (Fig 7D-2).

A negative correlation was also observed between *Pdu-miR396a* and its target Protein BRI1-5 ENHANCED 1 (BEN1), which is the major receptor of the plant brassinosteroid hormone that involved in hormone signaling and specifically increased at the final development of almond kernel (Fig 7E). The expression of *Pdu-miR396a* and BEN1 showed significant differences in different crosses of the studied cultivars. The highest and lowest expression of *Pdu-miR396a* determined 18 DAP of SM and 72 DAP for PM crosses, respectively (Fig 7E-1). 72 DAP of PM and 18DAP of PO had the highest and lowest level of BEN1 expression, respectively. No significant differences were observed between 0DAP, 18DAP and 36DAP crosses (Fig 7E-2).

## Discussion

Seed size is one of the most important parameters of plant fitness and the main component of the yield [45]. Various studies have been performed to know how plants control seed size in developmental biology. Seed development happens with a double-fertilization event in almond, which produces a diploid embryo and a triploid endosperm, as an angiosperm plant [46]. The seed coat covers the endosperm and the embryo, which is reached from female integuments. To ensure coordinated growth and development, all three seed structures are interconnected and thereby specifying a seed final size [47].

The seed size is influenced by genetic factors and environmental conditions. It is demonstrated that there are several factors that regulate endosperm growth and thus control seed size [48]. The genotype of zygotic tissues directly affects the seed size nor the genotype of maternal tissues [49]. For example, reciprocal crosses of two genotypes include the met1-6 mutant, a mutation of the MET1 gene, and wild type show that hypomethylated paternal and maternal genomes produce significantly smaller and larger F1 seeds, respectively [50]. In Almond, we study effect of pollen on seed size at the morphological and anatomical levels and the comparison of shell weight, kernel weight, kernel length and kernel diagonal content means showed that the pollination with Mamaee pollinizer increased the Shell weight, Kernel weight, Kernel length, Kernel diagonal and Kernel diameter kernel of two female parents. Also, comparison of cell area content means showed that the pollination with ‘Mamaee’ pollinizer increased the cell area kernel of two female parents. So that, the cross between ‘Mamaee Pooya’× ‘Mamaee’ (PM), and ‘Sefid’×‘Mamaee’ (SM) produced kernel with cell area larger than the cross between ‘Mamaee Pooya’×‘Orientalis’ (PO) and ‘Sefid’×‘Orientalis’ (SO), respectively.

So, we found some key gene in developing seeds of these crosses that control seed size through maternal and paternal genotypes [11]. In this research we used two composite samples to sequence the small RNAs for identification of miRNA and target genes to study the mechanism of seed development. The single composite sample analysis assayed using RNA-Seq is a limited approach [51] although this unreplicated treatment composed by a pool of fruits practically corrects this limitation. Additionally, with the use of qPCR this approach is completely validated using a pool of samples. Single composite sample RNA-Seq analysis has been applied in many studies including the effect of different pollination on the expression of Dangshan Su pear microRNA [34], plant-pathogen interactions in fungi [35, 52]; and also, in virus chenopodium susceptibility to different virus [53] and PPV susceptibility in peach [54]. In the same vein, partial validation of RNA-Seq data (read number) with the application of qPCR (expression level) in biological samples has been described in the analysis of plant-pathogen interactions in the case of fungi [52, 55] and viruses and viroids [53]. In these assays, a selected number of genes have been evaluated in different biological replications in order to validate the obtained RNA-Seq results. We observe different expression for some miRNAs and their targets (*Pdu-miR395a-3p*-NFYB3, *Pdu-miR8123-5p*-SPX1, *Pdu-miR482f*-PGSIP3 (GUX2), *Pdu-miR6285*-CH3.9, and *Pdu-miR396a*-BEN1) by integrated analysis of miRNA transcriptome and RT-qPCR in kernels with a different size. So we suggested that this miRNA effective on seed size through target genes.

Fruit maturation depends on the coordinated adjustment of many genes, such as ripening-related transcription factors (TFs), fruit-related microRNAs, DNA methylation and chromatin remodeling. Therefore, it is a complicated developmental process. The studies showed that several TFs, such as MADS-domain, MYB, AP2/ERF, and SBP/SPL family proteins play main roles in the adjustment of ripening. However, less attention has been paid to members of the large family in regard to fruit maturation, although genes in the NF-Y TF family are known to have important roles in regulating plant growth and development. For example, functional studies of gene expression suppression have shown that NF-Y genes influence fruit maturation by varying responses to ethylene when the fruits start to ripen [56]. In almond, the present study showed that the nuclear transcription factor Y Subunit B-3 (NFYB3) gene is the target of *Pdu-miR395a-3p* and the significant differential expression of this miRNA was observed in almond kernels of different sizes. The expression level of *Pdu-miR395a-3p* was found to be negatively correlated with seed size and its target gene was found to be positively correlated with seed size. Also the cross between female parent and male parent verifies the effect of NFYB3 gene on kernel size by male parent. These results indicating its role in increase of kernel growth by varying responses to ethylene therefore increase cellular expansion. Various studies have indicated that the expression level of *miR395a* is different significantly in developing fruit such as the seeds of Siberian apricot [17], pear fruit [18] and common bean seed [57]. Also, identification of known miR395 family in the stone-hardening stage of grape berries showed that involved in grape development and ripening process [33].

In accordance with differential expression of *miR8123* obtained from developing *Cajanus cajan* seeds [58], significant differences were observed in the expression of *Pdu-miR8123-5p* in almond kernel of varying sizes. Nithin et al. [58] predicted many targets for Cca-miR8123a such as mRNAs coding for chaperones, ribosomal proteins, transporters, kinases, signal transducers, ubiquitination proteins, receptors, and spliceosomal RNAs. Fan et al. [59] by integrated analysis of DE miRNAs and mRNAs showed that “amino acid metabolism”, “energy metabolism” and “lipid metabolism” were the most enriched under Pi deficiency. *Pdu-miR8123-5p* controlled the SPX1 (SPX domain-containing protein 1) target gene and regulated Pi transport and signaling in the developing kernel of almond. In the soil, the availability of nutrient solutions intensely affects plant growth and development. Among macro-elements, phosphorus (P) is the most essential and limiting one for plants. In plants, P deficiency is often happening because of its low concentration in the form of inorganic phosphate (Pi). However, plants have developed molecular mechanisms which are precisely controlled at the transcript and protein levels to increase P use efficiency to cope with these situations [60]. However, one of the main players which controls a set of processes involved in maintenance of an internal stable condition of phosphate ions at the cell level are proteins containing SPX domain, defined as Pi homeostasis [61, 62]. It has been revealed that the members of SPX family are expressed in many plant tissues including root, leave, cotyledon, stem and pollen [63]. The expression levels of some of the Phosphate Starvation Inducible (PSI) genes, such as PAP2, ACP5 and RNS1 were increased by overexpressing the AtSPX1, independent of Pi status, suggesting that AtSPX1 has a potential transcriptional regulation role on Pi starvation. TheOsSPX1 was suppressed by RNAi that cause reduction of plant growth and increased Pi accumulation in the shoots, as observed for well-characterized plants over accumulating Pi, such as Ospho2 mutants or OsPHR2-overexpressing plants [63]. In contrast, although growth was still reduced, overexpression of OsSPX1 suppressed the induction of the PSI genes, suggesting that OsSPX1, similar to AtSPX3, is involved in a negative feedback loop to adjust the expression of several PSI genes under Pi-limited conditions [63]. By using plants simultaneously overexpressing OsSPX1 and OsPHR2, Liu et al. [64] demonstrated that OsSPX1 could neutralize the function of OsPHR2 in inducing the expression of OsPT2, which plays a major role in Pi translocation and accumulation, as a result demonstrating that OsSPX1 acts as a negative regulator of OsPHR2. Lei et al. [65] showed the importance of sugars and, more specifically, sucrose in Pi homeostasis. The importance of Sucrose synthase (*SUS*) in starch production and sink strength determination in heterotrophic organs were supported by various studies including quantitative trait loci (QTL) analyses in maize endosperms and cotton [66] and the increase of biomass and fiber yield in SuSy-overexpressing cotton plants [67]. Therefore, the deciphering of the cross-talk between phosphate starvation and sugar signaling, as well as the involvement of SPX domain-containing proteins in these pathways on control seed size, requires further investigation.

In the present study, based on the recent findings of the key roles of the proteins harboring the SPX domain in phosphate signaling, we provide further research directions to improve our information on the nutrition of Phosphorus in plants, thus enabling the production of large size seeds. Also, the expression of SPX1 in developing almond seed revealed that they are under post-transcriptional control by *Pdu-miR8123-5p*, indicating the key role of miRNA-mediated regulation of SPX1 on Pi transport and signaling in developing seed. In this study, the SPX1 expression level was down-regulated in kernel tissues from the fruits with a large kernel (‘Mamaee Pooya’), also interaction between PM with PO and SM with SO show that this gene effective on kernel size via male parent. Thus, it is suggested that this gene exhibit its role in Pi translocation and accumulation levels and consequently reduce kernel size by an effect on Pi transport and signaling.

In plants, starch is the most abundant storage product which is synthesized in the plastid compartment (the chloroplast in photosynthetic cells or the amyloplast in non-photosynthetic cells). It is a polymer of glucose consisting of largely linear amylose and highly branched amylopectin, which are organized into a three-dimensional semi-crystalline granule [68–70]. Plant glycogenin-like starch initiation proteins (PGSIPs) involve in starch biosynthesis, accordingly, Chatterjee et al. [71] showed knockout of PGSIP1 expression in Arabidopsis results in a reduction of the starch quantity in leaves. This illustrates its critical role in starch biosynthesis. Also, Lakhwani et al. [32] identified miR482 mediated targeted genes involved in cell wall hydrolysis including expansion, chitinase and polygalacturonase (PG). So they showed that these miRNAs can be a part of the regulatory process involved in banana fruit ripening. Also, Carbone et al. [31] identified miR482 mediated targeted genes involved in Carboxylic acid metabolic process in ripening olive fruit. In accordance, we found significant differences observed between the large and small developing almond seeds for *Pdu-miRNA482f* and its target gene, PGSIPs, which has a major role on starch biosynthesis in seed development. So *Pdu-miRNA482f* mediates target PGSIP3gene to regulate seed growth and development. Also, expression of this target gene increased in PM and SM in comparison to PO and SO, respectively. Therefore, the different expression levels of this gene indicating that kernel size affected by male parent through starch biosynthesis. Similar roles of this gene have been described previously in Arabidopsis development [71] and the identified *PGSIP3* influences on kernel growth.

In this study, *Pdu-miRNA6285* showed significant differences between the large and small almond seeds. *Pdu-miR6285* with targeting GH3.9 (Gretchen Hagen 3) has a major role in plant development. The *Pdu-miR6285* significantly showed low expression levels in the large seeds, and its target had a high expression level. Amino acids bind to phytohormones and change their roles depending on the type of hormone and amino acid so that connecting amino acids to indole-3-acetic acid (IAA) inactivates the major plant hormone auxin. Also, the amino acid connected to IAA changes the fate of the conjugate. The connection of either aspartate or glutamate leads to the degradation of IAA. In contrast to IAA-Asp and IAA-Glu, the conjugates IAA-Ala and IAA-Val can be hydrolyzed back to the active free IAA form and provide inactive storage forms of the hormone [72]. Gretchen Hagen 3 acyl acid amido synthetases affected the regulating levels of phytohormones, such as IAA and JA. These proteins adjust the phytohormone signaling pathways responsible for plant growth, seed development, light signaling, drought response, and pathogen resistance [73]. Therefore, the different expression levels of this miRNA indicating that kernel size affected by male parent through adjust the phytohormone signaling pathways responsible for plant growth with targeting GH3.9.

*MiRNA396a* is identified as an effective miRNA in the growth and development process. The gene regulation factors are the target genes of this miRNA which encode putative transcription factors that are involved in the regulation of plant growth and development [74]. In contrast, the target gene of *Pdu-miRNA396a* is Ben1 (Brassinosteroid insensitive 1) in almond which is the major receptor of the plant brassinosteroid hormone. It plays very important roles in plant development, especially in the control of cell elongation [75]. Zhang et al. [21] showed that *miR396a* were down-regulated at ripening stages compared with the early stages of Hami melon fruit development. BRI1 enhances cell elongation, promotes pollen development, and controls vasculature development. In the present study, the expression of the Ben1 gene was higher in large seeds. Thus, suggested that *Pdu-miRNA396a* mediates Ben1 target gene to regulate seed growth and development of almond. So that Hwang et al. [76] reported that the signaling pathways of brassinosteroids (BRs) are critically in a various range of plant growth and developmental processes as well as many important agronomic traits. Also, effect of male parent on kernel size showed by higher expression of this target gene in PM and SM in comparison to the PO and SO, respectively. These results suggest that Ben1 gene influences on kernel size, so that yield is closely related to effect of Ben1 on cell elongation.

## Conclusion

Small RNA presence on almond kernel tissues could be easily sequenced and analyzed to identify important miRNAs involved in controlling kernel size. Since most of the commercial almond cultivars are self-incompatible and then need cross-pollination, for identifying kernel-size-related miRNAs and investigating the role of paternal effects on kernel size we have investigated different crosses between almond cultivars with small and large kernels. Furthermore, we retrieved the expression profile of predicted target genes obtained from a public database. Different expression profiles of target genes were observed among different crosses and developmental stages. An integrated analysis of miRNA transcriptome and RT-qPCR crosses of kernels with a different sizes allowed us to identify some key miRNAs and their targets (*Pdu-miR395a-3p*-NFYB3, *Pdu-miR8123-5p*-SPX, *Pdu-miR482f*-PGSIP3 (GUX2), *Pdu-miR6285*-GH3.9, and *Pdu-miR396a*-BEN1) potentially involved in regulating seed size of almond by maternal and paternal parents. In fact, sRNA-Seq and qPCR data confirmed that the expression of NYFB-3, SPX1 PGSIP3, GH3.9 and BEN1 genes have significant changes between combination and stages of development suggesting that should be involved in the kernel size because the presence of these miRNAs have a negative effect on their target genes. Further work, however, such as RNAi strategies and/ or overexpression studies are needed to confirm the accurate role of these genes in almond.

## Supporting information

S1 Table The list of primers used for miRNAs and target genes expression analysis by qPCR. (DOCX)

S2 Table The list of conserve miRNAs in some plant species. (XLSX)

S3 Table Description of miRNAs targeting gene. (XLSX)

S4 Table GLM of the miRNAs and target genes expression. (DOCX)

## References

[1] Kester DE, Asey R. Almonds. In ’Advances in Fruit Breeding’. Eds Janick J and Moore J. N. 1975; pp.387–412.

[2] Griggs WH. Pollination requirements of fruits and nuts. California Agriculture Station Service Circular. 1953; pp.424.

[3] Socias i Company R, Kester DE, Bradley MV. Effects of genotype and temperature on pollen tube growth in some self-incompatible and self-compatible almond cultivars. J Am Soc Hort Sci. 1976;101:490–3.

[4] Sánchez-Pérez R, Dicenta F, Martínez-Gómez P. Identification of S-alleles in almond using multiplex-PCR. Euphytica. 2004;138(3):263–269. doi: 10.1023/B:EUPH.0000047097.96271.bf

[5] Jackson JF. Gene flow in pollen in commercial almond orchards. Sex Plant Rep. 1996;9:367–369. doi: 10.1007/BF02441958

[6] Vezvaei A, Jackson JA. Effect of pollen parent and stages of flower development on almond nut production. Australian Journal of Experimental Agriculture. 1995;35:109–113. doi: 10.1071/EA9950109

[7] Hossein-Ava S, Imani A, Makhof M. An investigation of percentage of dichogamy and selection of the best pollinizer for commercial varieties of hazelnut. Int. J. Hort. Sci. Tech. 2006;37(2):370–380.

[8] Javadi D, Gheshlaghi AE. Effect of different pollen sources on nut and kernel characteristics of hazelnut (*Corylus avellana* L.). Int J Hort Sci Technol. 2006;7:15–22. https://www.sid.ir/en/journal/ViewPaper.aspx?id=57874.

[9] Sánchez-Pérez R, Arrázola G, Martín ML, Grané N, Dicenta F. Influence of the pollinizer in the amygdalin content of almonds. Sci Hort. 2012;139:62–65. doi: 10.1016/j.scienta.2012.02.028

[10] Crane JC, Iwakiri BT. Xenia and metaxenia in pistachio. Hort Sci. 1980;15:184–185.

[11] Jafari M, Shiran B, Rabiei Gh, Ravash R, Sayed Tabatabaei BE, Pedro MG. Identification of xenia effects and candidate genes linked to kernel size in almond [*Prunus dulcis* (Mill.) D.A. Webb] by using RNA-Seq.

[12] Phillips J, Dalmay T, Bartels D, Dalmay T, Bartels D, Bartels D. The role of small RNAs in abiotic stress. FEBS Letters. 2007;581:3592–3597. doi: 10.1016/j.febslet.2007.04.007 17451688

[13] Bartel DP. MicroRNAs: genomics, biogenesis, mechanism, and function. Cell. 2004;116:281–297. doi: 10.1016/s0092-8674(04)00045-5 14744438

[14] Jones-Rhoades MW, Bartel DP. Computational identification of plant microRNAs and their targets, including a stress-induced miRNA. Mol Cell. 2004;14:787–799. doi: 10.1016/j.molcel.2004.05.027 15200956

[15] Ori N, Cohen AR, Etzioni A, Brand A, Yanai O, Shleizer S, et al. Regulation ofLANCEOLATEby miR319 is required for compound-leaf development in tomato. Nat Genet. 2007;39:787–791. doi: 10.1038/ng2036 17486095

[16] Chen XM. A microRNA as a translational repressor of APETALA2 in Arabidopsis flower development. Science (Wash). 2004;303:2022–2025. doi: 10.1126/science.1088060 12893888 PMC5127708

[17] Niu J, Wang J, An J, Liu L, Lin Z, Wang R, et al. Integrated mRNA and miRNA transcriptome reveal a cross-talk between developing response and hormone signaling for the seed kernels of Siberian apricot. Sci Rep. 2016;6:35675. doi: 10.1038/srep35675 27762296 PMC5071837

[18] Wu J, Wang D, Liu Y, Wang L, Qiao X, Zhang S. Identification of miRNAs involved in pear fruit development and quality. BMC Gene. 2014;15:953. doi: 10.1186/1471-2164-15-953 25366381 PMC4233070

[19] Yao J‐L, Xu J, Cornille A, Tomes S, Karunairetnam S, Luo Z, et al. A microRNA allele that emerged prior to apple domestication may underlie fruit size evolution. Plant J. 2015;84:417–427. doi: 10.1111/tpj.13021 26358530

[20] Allen RS, Li J, Stahle MI, Dubroué A, Gubler F, Millar AA. Genetic analysis reveals functional redundancy and the major target genes of the ArabidopsismiR159 family. Proc Natl Acad Sci USA. 2007;104:16371–16376. doi: 10.1073/pnas.0707653104 17916625 PMC2042213

[21] Zhang H, Yin L, Wang H, Wang G, Ma X, Li M, et al. Genome-wide identification of Hami melon miRNAs with putative roles during fruit development. PLoS One. 2017;12(7). doi: 10.1371/journal.pone.0180600 28742088 PMC5524408

[22] Zhang YC, Yu Y, Wang CY, Li ZY, Liu Q, Xu J. et al. Overexpression of microRNA OsmiR397 improves rice yield by increasing grain size and promoting panicle branching. Nat Biotech. 2013;31:848–852. doi: 10.1038/nbt.2646 23873084

[23] Cao D, Wang J, Ju Z, Liu Q, Li S, Tian H, et al. Regulations on growth and development in tomato cotyledon, flower and fruit via destruction of miR396 with short tandem target mimic. Plant Sci. 2016;247:1–12. doi: 10.1016/j.plantsci.2016.02.012 27095395

[24] Zheng L, Zhang X, Zhang H, Gu Y, Huang X, Huang H, et al. The miR164-dependent regulatory pathway in developing maize seed. Mol Genet Genomics 2019;294:501–517. doi: 10.1007/s00438-018-1524-4 30607602

[25] Dhaka N, Sharma R. MicroRNA-mediated regulation of agronomically important seed traits: a treasure trove with shades of grey. Cri Rev in Biotech. 2021;41(4):594–608. doi: 10.1080/07388551.2021.1873238 33682533

[26] Liu Y, Wang L, Chen D, Wu X, Huang D, Chen L, et al. Genome-wide comparison of microRNAs and their targeted transcripts among leaf, flower and fruit of sweet orange. BMC Genomics. 2014;15:695. doi: 10.1186/1471-2164-15-695 25142253 PMC4158063

[27] Wang C, Han J, Liu C, Kibet KN, Kayesh E, Shangguan L, et al. Identification of microRNAs from Amur grape (*vitis amurensis Rupr.*) by deep sequencing and analysis of microRNA variations with bioinformatics. BMC Genomics. 2012;13:122. doi: 10.1186/1471-2164-13-122 22455456 PMC3353164

[28] Aryal R, Jagadeeswaran G, Zheng Y, Yu Q, Sunkar R, Ming R, et al. Sex specific expression and distribution of small RNAs in papaya. BMC Genomics. 2014;15:20. doi: 10.1186/1471-2164-15-20 24410969 PMC3916515

[29] Ge A, Shangguan L, Zhang X, Dong Q, Han J, Liu H, et al. Deep sequencing discovery of novel and conserved microRNAs in strawberry (*Fragaria*×*ananassa*). Physiol Planta. 2013;148:387–396. 10.1111/j.1399-3054.2012.01713.x. 23061771

[30] Kang C, Liu Z. Global identification and analysis of long non-coding RNAs in diploid strawberry *Fragaria vesca* during flower and fruit development. *BMC Genomics*. 2015;16:815. doi: 10.1186/s12864-015-2014-2 26481460 PMC4617481

[31] Carbone F, Bruno L, Perrotta G, Bitonti MB, Muzzalupo I, et al. Identification of miRNAs involved in fruit ripening by deep sequencing of *Olea europaea* L. transcriptome. PLOS ONE. 2019;14(8):e0221460. doi: 10.1371/journal.pone.0221460 31437230 PMC6705801

[32] Lakhwani D, Pandey A, Sharma D, Asif MH, Trivedi PK. Novel microRNAs regulating ripening-associated processes in banana fruit. Plant Growth Reg. 2020;90:223–235. 10.1007/s10725-020-00572-w.

[33] Wang C, Wang W, Abdelrahman M, Jiu S, Zheng T, et al. Study on miRNAs-Mediated Seed and Stone-Hardening Regulatory Networks and the Mechanism of miRNAs’ Manipulating Gibberellin-Induced Seedless Berries in Grapevine (*Vitis vinifera* L.). 2020. 10.21203/rs.3.rs-101517/PMC8480016 34587914

[34] Cheng X, Yan C, Zhang J, Ma C, Li S, Jin Q, et al. The effect of different pollination on the expression of Dangshan Su pear microRNA. BioMed research international. 2017. doi: 10.1155/2017/2794040 28497043 PMC5402243

[35] Brakat A, Staton M, Cheng CH, Park J, Yassin NM, Ficklin S, et al. Chestnut resistance to the blight disease: insights from transcriptome analysis. BMC Plant Biology. 2012;12(1):1–5. 22429310 10.1186/1471-2229-12-38 PMC3376029

[36] Bustin SA, Beaulieu JF, Huggett J, Jaggi R, Kibenge FSB, Olsvik PA, et al. MIQE précis: Practical implementation of minimum standard guidelines for fluorescence-based quantitative real-time PCR experiments. BMC Molecular Biol. 2010;11:74. doi: 10.1186/1471-2199-11-74 20858237 PMC2955025

[37] Rubio-Piña JA, Zapata-Pérez O. Isolation of total RNA from tissues rich in polyphenols and polysaccharides of mangrove plants. Electron. J. Biotech. 2011;14(5):11–18.

[38] Barakat A, Sriram A, Park J, Zhebentyayeva T, Main D, Abbott A. Genome wide identification of chilling responsive microRNAs in *Prunus persica*. BMC Genomics. 2012;13:481. doi: 10.1186/1471-2164-13-481 22978558 PMC3463484

[39] Cox MP, Peterson DA, Biggs PJ. SolexaQA: at-a-glance quality assessment of Illumina second-generation sequencing data. BMC Bioinformatics. 2010;11:485. doi: 10.1186/1471-2105-11-485 20875133 PMC2956736

[40] Gao Z, Shi T, Luo X, Zhang Z, Zhuang W, Wang L. High-throughput sequencing of small RNAs and analysis of differentially expressed microRNAs associated with pistil development in Japanese apricot. BMC Genomics. 2012;13:371. doi: 10.1186/1471-2164-13-371 . 22863067 PMC3464595

[41] Dai X, Zhao PX. psRNATarget: a plant small RNA target analysis server. Nucleic acids research. 2011;39 (suppl2):W155–W9. doi: 10.1093/nar/gkr319 21622958 PMC3125753

[42] Du Z, Zhou X, Ling Y, Zhang Z, Su Z. agriGO: a GO analysis toolkit for the agricultural community. Nucl Acids Res. 2010;38:W64–70. doi: 10.1093/nar/gkq310 20435677 PMC2896167

[43] Varkonyi-Gasic E, Wu R, Wood M, Walton EF, Hellens RP. Protocol: a highly sensitive RT-PCR method for detection and quantification of microRNAs. Plant Methods. 2007;3:12. doi: 10.1186/1746-4811-3-12 17931426 PMC2225395

[44] Karimi M, Ghazanfari F, Fadaei A, Ahmadi L, Shiran B, Rabei M, et al. The small-RNA profiles of almond (*Prunus dulcis* Mill.) reproductive tissues in response to cold stress. PLoS One. 2016;11(6):p.e0156519. doi: 10.1371/journal.pone.0156519 27253370 PMC4890778

[45] Alonso-Blanco C, Blankestijn-de Vries H, Hanhart CJ, Koornneef M. Natural allelic variation at seed size loci in relation to other life history traits of *Arabidopsis thaliana*. Proc Natl Acad Sci USA. 1999;96:4710–4717. doi: 10.1073/pnas.96.8.4710 10200327 PMC16397

[46] Chaudhury AM, Koltunow A, Payne T, Luo M, Tucker MR, Dennis ES. et al. Control of early seed development. Annu Rev Cell Dev Biol. 2001;17:677–699. doi: 10.1146/annurev.cellbio.17.1.677 11687501

[47] Figueiredo DD, Kohler C. Signalling events regulating seed coat development. Biochem Soc Trans. 2014;42:358–363. doi: 10.1042/BST20130221 24646244

[48] Luo M, Dennis ES, Berger F, Peacock WJ, Chaudhury A. MINISEED3 (MINI3), a WRKY family gene, and HAIKU2 (IKU2), a leucine-rich repeat (LRR) KINASE gene, are regulators of seed size in Arabidopsis. Proc Natl Acad Sci USA. 2005;102(48):17531–6. doi: 10.1073/pnas.0508418102 16293693 PMC1297679

[49] Sun X, Shantharaj D, Kang X, Ni M. Transcriptional and hormonal signaling control of Arabidopsis seed development. Curr Opin Plant Biol. 2010;13(5):611–620. doi: 10.1016/j.pbi.2010.08.009 20875768

[50] Xiao W, Brown RC, Lemmon BE, Harada JJ, Goldberg, RB, Fischer RL. Regulation of Seed Size by Hypomethylation of Maternal and Paternal Genomes. Plant Physiol. 2006;142(3):1160. doi: 10.1104/pp.106.088849 17012404 PMC1630758

[51] Auer PL, Doerge RW. Statistical design and analysis of RNA sequencing data. Genetics. 2010;185(2):405–16. doi: 10.1534/genetics.110.114983 20439781 PMC2881125

[52] Fernandez D, Tisserant E, Talhinhas P, Azinheira H, Vieira ANA, PETITOT AS, et al. 454‐pyrosequencing of *Coffea arabica* leaves infected by the rust fungus *Hemileia vastatrix* reveals in planta‐expressed pathogen‐secreted proteins and plant functions in a late compatible plant–rust interaction. Mol plant pathol. 2012;13(1):17–37. doi: 10.1111/j.1364-3703.2011.00723.x 21726390 PMC6638645

[53] Zhang Y, Pei X, Zhang C, Lu Z, Wang Z, Jia S, et al. De novo foliar transcriptome of Chenopodium amaranticolor and analysis of its gene expression during virus-induced hypersensitive response.2012.e45953. 10.1371/journal.pone.0045953 PMC3461033 23029338

[54] Rubio M, Rodríguez‐Moreno L, Ballester AR, de Moura MC, Bonghi C, Candresse T, et al. Analysis of gene expression changes in peach leaves in response to Plum pox virus infection using RNA‐S eq. Mol plant pathol. 2015;16(2):164–76. doi: 10.1111/mpp.12169 24989162 PMC6638525

[55] Bagnaresi P, Biselli C, Orrù L, Urso S, Crispino L, Abbruscato P, et al. Comparative transcriptome profiling of the early response to Magnaporthe oryzae in durable resistant vs susceptible rice (*Oryza sativa* L.) genotypes. PloS One. 2012;7(12):e51609. doi: 10.1371/journal.pone.0051609 23251593 PMC3520944

[56] Li S, Li K, Ju Z, Cao D, Fu D, Zhu H, et al. Genome-wide analysis of tomato NF-Y factors and their role in fruit ripening. BMC genomics. 2016;17(1):36. doi: 10.1186/s12864-015-2334-2 26742635 PMC4705811

[57] Parreira JR, Cappuccio M, Balestrazzi A, Fevereiro P, Araújo SDS. MicroRNAs expression dynamics reveal post-transcriptional mechanisms regulating seed development in *Phaseolus vulgaris* L. Hort Res. 2021;8(1):18. doi: 10.1038/s41438-020-00448-0 33436559 PMC7804330

[58] Nithin C, Thomas A, Basak J, Bahadur RP. Genome-wide identification of miRNAs and lncRNAs in *Cajanus cajan*. BMC genomics. 2017;18(1):878. doi: 10.1186/s12864-017-4232-2 29141604 PMC5688659

[59] Fan F, Shang X, Ding G. Zhou Z, Tan J. Integrated mRNA and miRNA Expression Analyses of *Pinus massoniana* Roots and Shoots in Long-Term Response to Phosphate Deficiency. J Plant Growth Regul.2021. doi: 10.1007/s00344-021-10335-0 33649694 PMC7905201

[60] Wang Y, Wang F, Lu H, Liu Y, Mao C. Phosphate uptake and transport in plants: an elaborate regulatory system. Plant and Cell Physiol. 2021.pcab011. 10.1093/pcp/pcab011. 33508131

[61] Lin SI, Santi C, Jobet E, Lacut E, El Kholti N, Karlowski WM, et al. Complex regulation of two target genes encoding SPX-MFS proteins by rice miR827 in response to phosphate starvation. Plant Cell and Physiol. 2010;51:2119–2131. doi: 10.1093/pcp/pcq170 21062869

[62] Kant S, Peng M, Rothstein SJ. Genetic regulation by NLA and MicroRNA827 for maintaining nitrate-dependent phosphate homeostasis in Arabidopsis. PLoS Genetics 7. 2011;e1002021. doi: 10.1371/journal.pgen.1002021 21455488 PMC3063762

[63] Wang Z, Hu H, Huang H, Duan K, Wu Z, Wu P. Regulation of OsSPX1 and OsSPX3 on expression of OsSPX domain genes and Pi-starvation signaling in rice. Journal of Integrative Plant Biology. 2009;51:663–674. doi: 10.1111/j.1744-7909.2009.00834.x 19566645

[64] Liu F, Wang Z, Ren H, Shen C, Li Y, Ling HQ, et al. OsSPX1 suppresses the function of OsPHR2 in the regulation of expression of OsPT2 and phosphate homeostasis in shoots of rice. Plant J. 2010;62:508–517. doi: 10.1111/j.1365-313X.2010.04170.x 20149131

[65] Lei M, Liu Y, Zhang B, Zhao Y, Wang X, Zhou Y, et al. Genetic and genomic evidence that sucrose is a global regulator of plant responses to phosphate starvation in Arabidopsis. Plant Physiol. 2011;156:1116–1130. doi: 10.1104/pp.110.171736 21346170 PMC3135933

[66] Thévenot C, Simond-Côte E, Reyss A, Manicacci D, Trouverie J, le Guilloux M, et al. QTLs for enzyme activities and soluble carbohydrates involved in starch accumulation during grain filling in maize. J of Exp Bot. 2005;56(413):945–958. doi: 10.1093/jxb/eri087 15710637

[67] Xu SM, Brill E, Llewellyn DJ, Furbank RT, Ruan YL. Overexpression of a potato sucrose Synthase gene in cotton accelerates leaf expansion, reduces seed abortion, and enhances fiber production. Molecular Plant. 2012;5(2):430–441. doi: 10.1093/mp/ssr090 22115917

[68] James MG, Denyer K, Myers AM. Starch synthesis in the cereal endosperm, Curr Opin *Plant Biol*. 2003;6:215–222. doi: 10.1016/s1369-5266(03)00042-6 12753970

[69] Martin C, Smith AM. Starch biosynthesis. The Plant Cell. 1995;7(7):971–985. doi: 10.1105/tpc.7.7.971 7640529 PMC160895

[70] Zeeman SC, Kossmann J, Smith AM. Starch: its metabolism, evolution, and biotechnological modification in plants. *Annual review of plant biology*, 2010;61:209–234. doi: 10.1146/annurev-arplant-042809-112301 20192737

[71] Chatterjee M, Berbezy P, Vyas D, Coates S, Barsby T. Reduced expression of a protein homologous to glycogenin leads to reduction of starch content in Arabidopsis leaves. Plant Sci. 2005;168(2):501–509. doi: 10.1016/j.plantsci.2004.09.015

[72] LeClere S, Tellez R, Rampey RA, Matsuda SP, Bartel B. Characterization of a family of IAA-amino acid conjugate hydrolases from Arabidopsis. J Biol Chem. 2002;277(23):20446–20452. doi: 10.1074/jbc.M111955200 11923288

[73] Westfall CS, Sherp AM, Zubieta C, Alvarez S, Schraft E, Marcellin R, et al. Arabidopsis thaliana GH3. 5 acyl acid amido synthetase mediates metabolic crosstalk in auxin and salicylic acid homeostasis. Proc Natl Acad Sci USA. 2016;113(48):13917–13922. doi: 10.1073/pnas.1612635113 27849615 PMC5137743

[74] Correa JP, de O’Silva EM, Nogueira FTS. Molecular Control by non-coding RNAs during fruit development: from gynoecium patterning to fruit ripening. Front Plant Sci. 2018;9:1–13. doi: 10.3389/fpls.2018.00001 30555499 PMC6283909

[75] Jiang WB, Lin WH. Brassinosteroid functions in Arabidopsis seed development. Plant Signal Behav. 2013;8(10):e25928. doi: 10.4161/psb.25928 24270689 PMC4091071

[76] Hwang H, Ryu H, Cho H. Brassinosteroid Signaling Pathways Interplaying with Diverse Signaling Cues for Crop Enhancement. Agronomy. 2021;11:556.

