# Supplementary material for: Identification and verification of seed development related miRNAs in kernel almond by small RNA sequencing and qPCR
Source: PLoS One. 2021 Dec 1;16(12):e0260492. doi: 10.1371/journal.pone.0260492 (PMC8635354; doi:10.1371/journal.pone.0260492)
Supplement: S1 Table — (DOCX) [file pone.0260492.s001.docx]

Table S1. The list of primers used for miRNA and target gene expression analysis by quantitative Real Time PCR. Corresponding annealing temperatures are shown for each gene. Abbreviations: F, Forward primer; R, Reverse primer.

| Primer name | Primer sequences 5′->3′ | Annealing (°C) |
| --- | --- | --- |
| Pdu-mir395a | F: GTTGCTGAAGTGTTTGGGG | 57 |
|  | R:GTTGGCTCTGGTGCAGGGTCCGAGGTATTCGCACCAGAGCCAAC GGGTCC | 89 |
| Pdu-mir482f | F: GTGTCTTTCCTACTCCACC | 57 |
|  | R:GTTGGCTCTGGTGCAGGGTCCGAGGTATTCGCACCAGAGCCAAC GGAATG | 87 |
| Pdu-mir6285 | F: GTGGGGTAGTGAAGTTTGAATT | 58 |
|  | R:GTTGGCTCTGGTGCAGGGTCCGAGGTATTCGCACCAGAGCCAAC AGCCCT | 88 |
| Pdu-mir8123 | F: GTTGTGAGCAATGGCACAC | 57 |
|  | R:GTTGGCTCTGGTGCAGGGTCCGAGGTATTCGCACCAGAGCCAAC AGGGCT | 88 |
| Pdu-mir396a | F: GGGGTTCCACAGCTTTCTT | 57 |
|  | R:GTTGGCTCTGGTGCAGGGTCCGAGGTATTCGCACCAGAGCCAAC ACGTTC | 87 |
| universal | GTGCAGGGTCCGAGGT | 56 |
| Pdu-PGSIP3 | F: TATGCCACCAAATGCTTCCG | 58.90 |
|  | R: TGCAGAGGTTTTGTGGGTTG | 58.89 |
| Pdu-GH3.9 | F: TTGTGCCGTTGTTGAAAGGG | 59.54 |
|  | R: TCAACACCTTGTGGTTTGCC | 59.18 |
| Pdu-NFYB3 | F: TTCAGCAGTGATGGTCTTGC | 58.47 |
|  | R: ACGATGCCAAGGAAACCATC | 58.54 |
| Pdu-SPX1 | F: TCTCCAGCAAAACCA TCTCTCC | 60.03 |
|  | R: ACAGGGTGGCAAAAGCAAAG | 59.53 |
| Pdu-BEN1 | F: TCACAAGGCTGCTGCTTTTG | 59.61 |
|  | R: ACTCCAAGACAGTGAAACGC | 58.42 |
