# Supplementary material for: Identification and verification of seed development related miRNAs in kernel almond by small RNA sequencing and qPCR
Source: PLoS One. 2021 Dec 1;16(12):e0260492. doi: 10.1371/journal.pone.0260492 (PMC8635354; doi:10.1371/journal.pone.0260492)
Supplement: S4 Table — (DOCX) [file pone.0260492.s004.docx]

Table S4: GLM of the miRNAs and target genes expression

| source | df | miR395a-3p | miR6285 | miR8123-5p | miR396a | miR482f | NYFB-3 | BEN1 | GH3,9 | PGSIP3 (GUX2) | SPX1 |
| --- | --- | --- | --- | --- | --- | --- | --- | --- | --- | --- | --- |
| block | 3 | 25.46* | 24.43** | 0.182^ns^ | 7.81** | 13.62** | 1272.79^ns^ | 2046.35** | 10.006** | 22.0^ns^ | 32.47** |
| cross | 3 | 4.74^ns^ | 46.93** | 7.89** | 8.67** | 2.57* | 1517.71* | 4268.17** | 432.99** | 845.02** | 10.14^ns^ |
| stage | 4 | 70.55** | 66.23** | 26.62** | 33.75** | 50.27** | 46904.17** | 23291.62** | 135.09** | 9166.51** | 76.44** |
| cross× stage | 12 | 6.096* | 7.35** | 1.07^ns^ | 1.9* | 1.67* | 1110.62* | 1513.05** | 25.308** | 184.11** | 5.68^ns^ |
| error | 57 | 2.75 | 0.602 | 0.846 | 0.779 | 0.838 | 541.5 | 284.45 | 0.711 | 51.16 | 4.32 |

^ns^: non-significant.

^**^ Significant at (P<1%)

^*^ Significant at (P<5%)
